# Designing A Transgenic Chicken: Applying New Approaches toward A Promising Bioreactor

**DOI:** 10.22074/cellj.2020.6738

**Published:** 2019-10-14

**Authors:** Salahadin Bahrami, Amir Amiri-Yekta, Abbas Daneshipour, Seyedeh Hoda Jazayeri, Paul Edward Mozdziak, Mohammad Hossein Sanati, Hamid Gourabi

**Affiliations:** 1. Department of Genetics, Reproductive Biomedicine Research Center, Royan Institute for Reproductive Biomedicine, ACECR, Tehran, Iran; 2. Physiology Graduate Program, North Carolina State University, Raleigh, NC, USA; 3. Department of Medical Genetics, National Institute of Genetic Engineering and Biotechnology, Tehran, Iran

**Keywords:** Chickens, CRISPR/Cas9, Ovalbumin, Recombinant Protein, Transgenes

## Abstract

Specific developmental characteristics of the chicken make it an attractive model for the generation of transgenic organisms. Chicken possess a strong potential for recombinant protein production and can be used as a powerful bioreactor to produce pharmaceutical and nutritional proteins. Several transgenic chickens have been generated during the last two decades via viral and non-viral transfection. Culturing chicken primordial germ cells (PGCs) and their ability for germline transmission ushered in a new stage in this regard. With the advent of CRISPR/Cas9 system, a new phase of studies for manipulating genomes has begun. It is feasible to integrate a desired gene in a predetermined position of the genome using CRISPR/Cas9 system. In this review, we discuss the new approaches and technologies that can be applied to generate a transgenic chicken with regards to recombinant protein productions.

## Introduction

The first genetically modified chicken was reported in 1989 (1, 2) and thereafter many other transgenic avian species have been generated, with special attention to chicken and quail (2). These transgenic species possess a great potential for many purposes, including the poultry industry, medicine and drug manufacturing, developmental studies, research and investigation of disease susceptibility and creating biomedical models for different scientific purposes (2- 4). Many scientists around the world have focused on exploiting transgenic technology to generate transgenic chicken for a practice known as biopharming, since the chicken egg is used as a preferential bioreactor to produce pharmaceutical and nutritional proteins (5, 6). The most interesting aspect of this new technology is the potential to produce therapeutic recombinant proteins in large quantities. The market demand for some of these recombinant proteins (for example monoclonal antibodies) are high; so a high producing system is required (7).

Overall, transgenic animals and transgenic chicken, in particular, represent a great potential for production of therapeutic recombinant proteins, because they have the ability to produce very complex and active proteins while at the same time providing the appropriate posttranslational modifications (8, 9). An inability to provide the appropriate translational modifications is the most important drawback of bacterial bioreactors (10), the most cost-effective system. Transgenic animals are superior (9) to transgenic plants (11, 12) and insects which have a relatively slow production setup (9). With regard to production cost, a transgenic animal farm is much more cost-effective than building a large-scale manufacturing facility for culturing mammalian cells (13, 14) which Dyck et al. (15) estimates would likely cost over five times more than that needed to produce transgenic animals. The time and expense needed for chicken to reach maturity and begin to produce target compounds is much less than those needed with other farm animals. Although chicken pose the risk of zoonotic diseases, the risk can almost be eliminated by using a closed rearing system and well established specific-pathogen-free (SPF) protocols. For the reasons covered above, chicken seems to be the best farm animal to be used as a transgenic bioreactor (16).

Egg white provides a promising substrate where a protein of interest can be accumulated in large amounts and subsequently be easily harvested for purification. The ovalbumin promoter facilitates localized production of ovalbumin, the main protein in egg white. This promoter can be modified to regulate production of a gene of interest (GOI) in oviduct cells in which the egg white is produced (17). In this short review, we explain the possibility of applying these new technologies in generating transgenic chicken. At the end, we describe a promising new strategy for generating a transgenic chicken which does not require insertion of an exogenous promoter in the construct.

### Methods for introducing the gene construct

Traditionally, DNA microinjection into the pronucleus of a freshly fertilized egg is used as the method of choice in order to introduce the genes in mammalian transgenesis (18, 19). However, microinjection cannot be easily applied to chicken, because there are more than 50000 cells in a freshly-laid fertilized chicken egg (20, 21). For this reason, microinjection can only be used on early stage fertile embryos collected from sacrificed hens (22, 23). Using this method, Love et al. (22) were able to generate a mosaic transgenic rooster carrying the lacZ gene. However, the offspring never expressed the protein. Even though microinjection can be successful, it is a slow and inefficient method for creating transgenic chicken. In addition, every time microinjection is performed, a hen must be sacrificed to collect the fertilized eggs (21). Furthermore, even in cases of successfully generated transgenic chicken, the desired protein production may not be achieved due to gene silencing or a positional effect of the gene (7, 24).

An alternative to microinjection is transfection of an exogenous gene done by using non-viral vectors or viral vectors. In most cases using non-viral vectors, the DNA construct is lost after multiple cell divisions, because it is not integrated into the host chromosome (25). Applying viral vectors is the most successful method (7, 21, 26), because the DNA construct naturally integrates into the host chromosomes. In fact, avian retroviral vectors derived from avian retroviruses were used to generate the first genetically modified chicken. The retroviral vectors were injected adjacent to the blastoderm which led to somatic mosaicism in 25% of samples and germinal transmission at rates of 1-11% (27). Since then, multiple scientific groups have applied different viral vectors to create transgenic chickens (1, 21). One drawback of this viral method is that the size of construct these vectors can carry is limited.

Modern retroviral vectors used to create transgenic chicken are replication-defective; the vector construct entails the least possible amount of viral sequence such as long terminal repeat (LTR) and a packaging signal, but not the viral genes essential for packaging gag, pol and env which are removed and replaced by the desired genetic sequences. To produce the virus particle, the vector containing the desired DNA construct is transfected into packaging cells such as HEK293 that produce gag/pol and env proteins. Subsequently, the virus particles are obtained from the culture supernatant. These viral particles can infect host cells and introduce their DNA along with the exogenous construct into the genome. However, in the absence of packaging genes, infection cannot create viral particles in the host cells. Thus, the integrated DNA will remain in the host genome and the transgene will probably have a stable expression (7).

Similar to the other animals, genomes of the avian species are prone to gene silencing (24) which is mainly associated with DNA methylation and it is transmitted to progeny (28). Histone modification and presence of the other chromatin condensing proteins can also cause silencing (7). By changing the timing of viral infection, Kamihira et al. (26) were able to overcome this problem and achieve the desired gene expression. When using viral vectors, the position of gene integration is random. Consequently, there is a strong possibility that transgene may integrate into a location which causes gene silencing. In addition, the gene integration may cause gene disruption in the host. As a result, there is a universal concern about the safety of this method (29).

### Primordial germ cells as the main target for transgenesis in chicken

Primordial germ cells (PGCs) are gamete progenitor cells, a population of undifferentiated cells that is separated from all somatic cells during early development. Unlike the PGCs in other species, PGCs in avian and some reptilian species migrate to the genital ridge via blood circulation. In the genital ridge, these PGCs are subjected to a series of complex processes which results in differentiation into functional spermatozoa or ova (30). PGCs have been the focus of researchers around the world and they have been widely used in manipulation of avian embryos (26, 31, 32). A conventional method to generate a transgenic chicken is to inject a high titer of the viral vectors into the subgerminal cavity of the embryos at stage X; so that the virus particles transfect the blastoderm cells along with PGCs (26, 31). In another method, the migrating PGCs are targeted by injecting a vector into the vascular system or directly injecting it into the heart of developing embryos after 50 to 60 hours of incubation (26). In fact, the first chimeric chicken was generated by Tajima et al. (33) by transplanting 100 chicken PGCs into a recipient embryo. Even though direct injection of PGCs successfully generated transgenic chicken, the process proved to be difficult, because it created a mosaic of PGCs in which only a small portion of them were transfected. Consequently, a time-consuming process is required to obtain a transgenic chicken. For this reason, researchers have spent a great deal of time trying to extract and enrich PGCs *in vitro* for subsequent manipulation. These enriched and transfected PGCs can be injected into a recipient embryo at the blastodermal stage or injected intravascularly between stages 13 and 16 thereby allowing them to migrate directly to the genital ridge (30, 31). Chicken PGCs were cultivated *in vitro*, for 4 days, for the first time in 1995 by Chang et al. (34). Kuwana et al. (35), Naito et al. (36) developed a PGC culture using a KAv-1 medium. In 2006, Van de Lavoir et al. (37) successfully cultivated male chicken PGCs *in vitro* and maintained the culture for over 100 days. In 2015, Whyte et al. (38) further improved culture conditions and proved that low osmotic pressure (up to 250 mosm/kg) and low calcium concentrations (up to 0.15 mM) were the best conditions for *in vitro* culture of chicken PGCs. This culture condition can maintain PGCs *in vitro* for a long period, so that the DNA manipulations can be achieved easily and transfected cells can be selected and enriched properly.

### Surrogate egg shell creates two windows of opportunity to manipulate chicken embryo

Different methods have been employed over the years to access the embryo in order to introduce foreign DNA: shell windowing, *ex vivo* embryo culturing and surrogate egg shell. In shell windowing a narrow window, about 20 mm in diameter, is opened at the blunt end of the egg providing easy access to the embryo, so manipulation can be achieved. Afterward, the window can be sealed with cling-film wrap and thin ovalbumin as a paste (29, 39, 40).

*Ex vivo* embryo culturing is the external culturing of a chicken embryo in conditions similar to that of the natural environment inside an egg. The method is thoroughly explained by Nakamura (29). In brief, the fertilized chicken egg and the thick surrounding albumin (8-16 ml) layer is collected from a hen and cultured in a sealed cup for one day at 41-42˚C(system I). The cultured embryo is then transferred to a surrogate shell filled with thin ovalbumin and tightly sealed (system II). After three days, the embryo is transferred to a larger, actual host egg with an empty space above the embryo such as a turkey egg shell (system III). This method provides windows of opportunity in which embryo manipulation can be easily performed which makes creating a transgenic chicken more practical.

In surrogate egg shell, the method includes two sequential transfers of the fertilized egg to different shells that correspond with system II and system III of the exvivo embryo culturing method (21, 41-43). In brief, the freshly laid fertile egg is transferred to an actual, slightly heavier egg shell (3-4 g), and the shell is filled with thin ovalbumin and sealed tightly with cling-film and ovalbumin paste (system II). After three days, the embryo is transferred to a bigger egg shell (fresh turkey or two yolk egg shell; 35-40 g), and the shell is sealed with cling-film and ovalbumin paste, while an empty space is provided above the embryo to expose the extra-embryonic membrane vascular system to the atmosphere. With this process, the embryo is accessible, but the system I of ex vivo embryo culturing process is not necessary, which makes it easier to perform.

### Applying CRISPR/Cas9-mediated targeted genome editing to chicken transgenesis

As it was mentioned above, exploiting germ cells such as PGCs provides an opportunity to transfect these cells, select the transfected ones, enrich them and subsequently inject these cells into a recipient embryo to generate transgenic chickens. To render a high and stable expression of a transgene, it is very important to ensure that the gene construct integrates into a position in the host genome that avoids gene silencing. Previously, positional targeting was pursued using homologous recombination vectors entailing homology regions of about 7-8 kb and worked with approximately 30% efficiency (32, 44). The problem with homologous recombination was the low efficiency of obtaining and cloning these long homology regions. Recent methods applying site-specific endonucleases such as Zinc finger nucleases (ZFNs) (45) and transcription activator-like effector nucleases (TALENs) (2, 46) have improved efficiency of the targeting approaches and consequently made them more popular. Despite their high efficiency, these endonucleases have limited use, because the construct design is very difficult and acquiring the desired endonuclease is not feasible in many cases. Moreover, the off-target rates are high (47).

**Fig 1:**
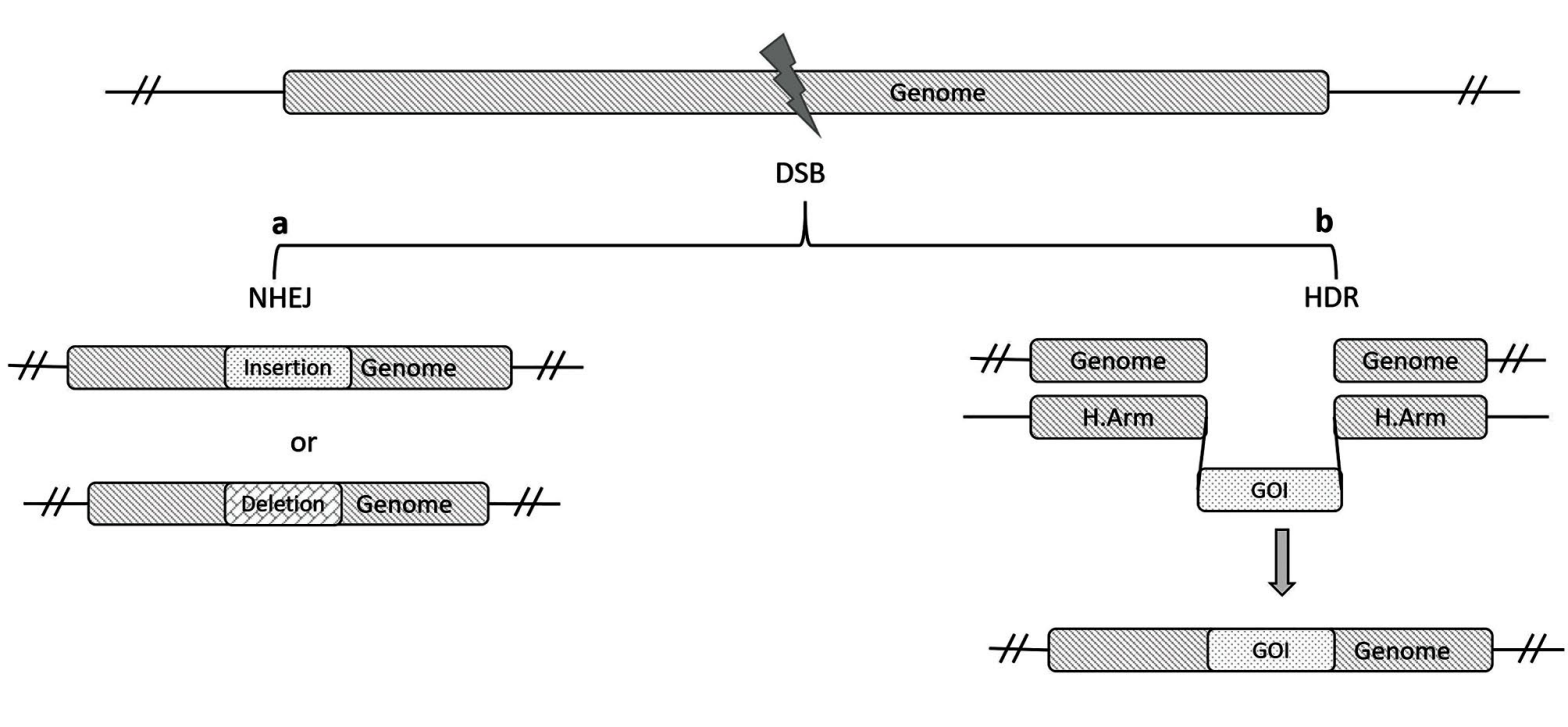
A schematic presentation of the double-strand break (DSB) repair. a. Non-homologous end joining (NHEJ) which directly ligates the DSB and can create insertions and deletions and b. Homology directed repair (HDR) in which a template DNA complementary to the break site is present.

In contrast, a recently emerged system, the clustered regularly interspaced short palindromic repeats (CRISPR)/ CRISPR-associated (Cas) system, has rendered a high success rate (80%), with much simpler construct designs (48). In this system, CRISPR-associated protein 9 (Cas9), the DNA endonuclease enzyme, is guided by a 20 bp RNA (gRNA) which pairs with the target DNA site. Other than the gRNA, a short protospacer adjacent motif (PAM) is required to ensure the complete interaction between Cas9 and the target DNA (49, 50). When the target DNA is complementary to the gRNA, Cas9 cleaves the DNA and creates a double-strand break (DSB) which can be repaired by either non-homologous end joining (NHEJ) or homology-directed repair (HDR). NHEJ may lead to small insertions/deletions whereas HDR is used when a template DNA complementary to the break site is present (51, 52) (Fig .1).

Oishi et al. (53) successfully applied CRISPR/Cas9 technology and efficiently (>90%) created mutations in two egg white genes, ovalbumin and ovomucoid, in cultured chicken PGCs which were subsequently injected into recipient chicken embryos. Zuo et al. (54) demonstrated that gene knockouts can be induced in both chicken stem cells and chicken embryos using CRISPR/ Cas9 technology. Using the CRISPR/Cas9 system, researchers successfully inhibited the chicken embryonic stem cells differentiation (ESCs) into spermatogonial stem cells (SSCs) by Stra8 gene knockdown (55). In another study, Dimitrov et al. (32) reported a successful gene editing in chicken PGCs using the CRISPR/ Cas9 system and a donor vector for HDR of the DSB. Recently, many scientists have applied this technology to generate gene knock-in in mammalian cells (56-58). With the CRISPR/Cas9 system, it is now possible to introduce a large DNA construct, which can entail a transgene into a specific locus in different cell lines (47, 48, 58, 59).

### Tissue-specific ovalbumin promoter: the best candidate for recombinant protein production in chicken

Chicken ovalbumin (OVA), the main protein in egg white, accounts for almost 55% of the total protein and is expressed strictly in oviduct cells. This gene is a well-known promoter with a very high expression ability which has been thoroughly studied as a model for tissue-specific expression (60, 61). Since the ovalbumin gene promoter is a tissue-specific promoter and is thought to have powerful production ability, it has been used to generate transgenic chickens with oviduct-specific production (5, 17, 62, 63). Four DNase I-hypersensitive sites (DHSs) have been identified in the 8.7 kb region between the ovalbumin gene and the Y gene, that is thought to be the regulation elements of the ovalbumin promoter (61, 64). The region is difficult to include entirety in a vector construct, because it is a large DNA sequence. As a result, researchers have investigated the role of DNase I hypersensitive sites, included fragments of the region as the promoter of choice (17, 43, 61), and reported oviduct tissuespecific expression of the transgene. Lillico et al. (62) demonstrated that 2.8 kb of the ovalbumin promoter, which encompasses a steroid-dependent regulatory element (SDRE) and a negative regulatory element (NRE), can strongly drive the transgene expression in oviduct cells. Liu et al. (43) used the same promoter to drive transgene expression inserted in different locations in the chicken genome. Their results showed different levels of expression, all lower than those in the previous study. These studies showed that location of the inserted transgene can significantly affect the expression level thereby emphasizing the importance of the positional effect of the insertion locus. They also indicated the necessity of the larger promoter region to maintain strong tissue-specific protein production, and that there may be other factors close to ovalbumin promoter contributing to its strong and tissue-specific expression.

### Applying new approaches

The most effective approach to produce transgenic chicken is to transfect PGCs *in vitro*, select the transfected cells and enrich them. Next, inject the cells into the circulating blood of an embryo or directly into the blastodisc. Using the CRISPR/Cas9 system, it is possible to integrate the DNA construct entailing the transgene into a previously determined position in the genome that ensures availability of the transgene and its favorable expression. As mentioned above, the ovalbumin promoter is one of the most interesting promoters which can regulate gene expression in the oviduct cells and later to the egg white. By using the CRISPR/Cas9 system and providing the homologous arms to induce HDR in the break site, a gene knock-in can be achieved *in vitro* using PGCs as the host cell. In 2015, Rojas-Fernandez et al. (65), successfully integrated a gene construct (firefly luciferase cDNA) downstream of an endogenous promoter (promoter of the TGFâ-responsive gene PAI-1) and demonstrated that the firefly luciferase cDNA expression mimicked that of endogenous PAI-1 expression. Consequently, it is feasible that an exogenous cDNA can be placed downstream of the endogenous ovalbumin promoter. In a recent work conducted by Oishi et al. (66), human interferon beta was inserted into the chicken ovalbumin locus. They created a CRISPR/Cas9-mediated knock-in of hIFN-â gene at the ovalbumin start codon located in exon 2 of the ovalbumin gene. The result demonstrated a promising transgene production in the egg white. It is plausible that the exon 1 of ovalbumin gene is a good candidate position to integrate the transgene.

**Fig 2:**
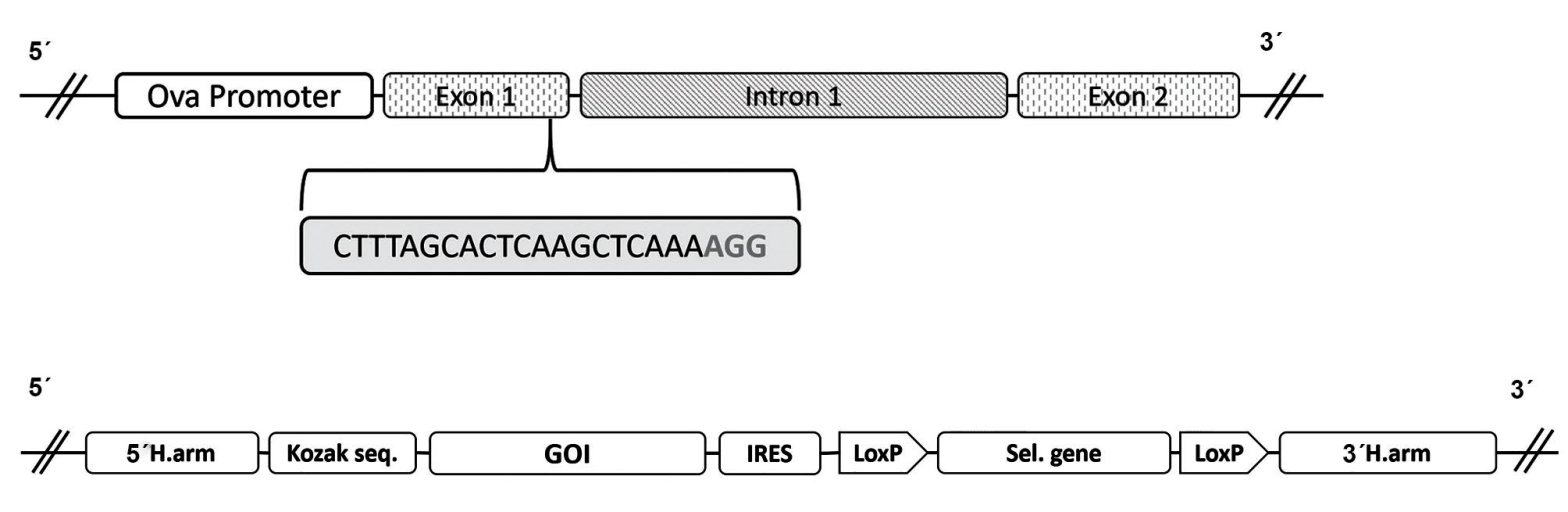
A schematic presentation of the candidate position and required donor vector for targeted integration. a. The ovalbumin gene, ova promoter, exons 1 and 2, as well as intron 1 is showed with the location of candidate gRNA and b. A schematic presentation of the vector entailing GOI with Kozak sequence and selectable gene flanked by homologous arms complementary to the DNA break site that can be induced by CRISPR.

The ovalbumin gene consists of eight exons and seven introns, and the start codon is located in exon 2. Using E-CRISPR software (67) and CRISPOR online software (68) analysis of the ovalbumin gene reveals a number of potential gRNA sites. The most promising one is CTTTAGCACTCAAGCTCAAAAGG which shows a high target affinity, high efficiency and a low off-target score (Fig .2A). Moreover, this gRNA site is located within the exon 1 which is not part of final ovalbumin cDNA and is not translated, so integrating an exogenous sequence in this location probably would not disrupt the ovalbumin gene and its splicing process. With this gRNA, the Cas9 nuclease will cut the DNA between C and A nucleotides close to the PAM sequence (AGG). The flanking 5' and 3' sequences around the break site can be used as homologous arms and add to corresponding terminals of the desired DNA construct (Fig .2B). Finally, adding a Kozak sequence (69) at the 5' end of gene construct, before the translation start codon, will ensure mRNA translation of the transgene. With this approach of removing promoter from the gene construct, more DNA sequence can be added to the vector (Fig .2A). A reporter gene or a selectable marker gene can be added to the 3' of the GOI using IRES sequence flanked by two LoxPs which can later be excised from the genome using Cre recombinase (70). Chicken PGCs can be transfected *in vitro* with a CRISPR/Cas9 vector and the DNA construct containing the GOI flanked with homologous arms and relative sequences. Transfected PGCs can be enriched and injected into chicken embryos to produce chickens with transgenic germ cells. Pure transgenic chickens producing the GOI in their egg white can then be achieved by breeding.

## Conclusion

Transgenic chicken provides a great opportunity to produce therapeutic proteins in large-scale, in both a timely and cost effective manner. However, developing a practical procedure to generate transgenic chicken proved to be challenging due to specific developmental characteristics of birds. Unlike mammals, a fertilized avian egg cannot be accessed in order to introduce DNA via microinjection, because a freshly laid chicken egg already contains more than 50000 cells. Several alternative methods have been developed and improved over the last decades to produce transgenic chicken, however, few successful cases were reported. Successful culturing of PGCs created a promising opportunity to manipulate these cells *in vitro*. With the advent of CRISPR/Cas9 system, it is now feasible to insert a GOI in a specific location of genome. Establishing a process to create transgenic chicken by inserting a foreign gene in a specific location where the exposure and expression of the gene are ensured, seems more possible than ever. As a result, great progress has already been achieved towards the goal of producing pharmaceutical or nutritional proteins with the creation of transgenic chickens producing a GOI in their egg white.
